# Fabrication Technology and Characteristics of a Magnetic Sensitive Transistor with nc-Si:H/c-Si Heterojunction

**DOI:** 10.3390/s17010212

**Published:** 2017-01-22

**Authors:** Xiaofeng Zhao, Baozeng Li, Dianzhong Wen

**Affiliations:** School of Electronic Engineering, Heilongjiang University, Harbin 150080, China; 2151246@s.hlju.edu.cn (B.L.); wendianzhong@hlju.edu.cn (D.W.)

**Keywords:** nc-Si:H/c-Si heterojunction, magnetic sensitive transistor, MEMS technology, temperature characteristics

## Abstract

This paper presents a magnetically sensitive transistor using a nc-Si:H/c-Si heterojunction as an emitter junction. By adopting micro electro-mechanical systems (MEMS) technology and chemical vapor deposition (CVD) method, the nc-Si:H/c-Si heterojunction silicon magnetically sensitive transistor (HSMST) chips were designed and fabricated on a p-type <100> orientation double-side polished silicon wafer with high resistivity. In addition, a collector load resistor ($R_{L}$) was integrated on the chip, and the resistor converted the collector current ($I_{C}$) to a collector output voltage ($V_{\mathrm{out}}$). When $I_{B}$ = 8.0 mA, $V_{\mathrm{DD}}$ = 10.0 V, and $R_{L}$ = 4.1 kΩ, the magnetic sensitivity ($S_{V}$) at room temperature and temperature coefficient ($\alpha_{C}$) of the collector current for HSMST were 181 mV/T and −0.11%/°C, respectively. The experimental results show that the magnetic sensitivity and temperature characteristics of the proposed transistor can be obviously improved by the use of a nc-Si:H/c-Si heterojunction as an emitter junction.

## 1. Introduction

In 1957, Kroemer presented a heterojunction structure transistor. Compared with the homojunction, by changing the band gap and carrier transmission via structural design, it is possible to improve the properties of a semiconductor device. In 2012, Tsai et al. proposed an InP/InGaAs heterojunction transistor, achieving a direct current (DC) voltage gain (*β*) of 255 [1]. In 2013, Narang et al. fabricated a Ga_0.5_In_0.5_P/GaAs heterojunction transistor to realize a DC voltage gain (*β*) of 100–120 [2]. In 2015, Saha et al. devised high-speed Si/SiGe heterojunction transistors, and the base transit time of heterojunction bipolar transistors reached 3.5 ps [3]. The above analysis demonstrates that the injection ratio of the heterojunction structure is higher than that of the homojunction structure, which is necessary to enhance voltage gain for ordinary transistors and to improve device characteristics [4,5].

In recent years, various fabrication techniques have been used to make magnetic sensing element in SiO_2_. Kennedy et al. proposed the use of ion implantation techniques to fabricate magnetic nanoclusters on SiO_2_-Si for magnetic sensor applications [6], and the current-voltage characteristics of magneto-resistance were investigated [7]. In the meantime, using the techniques of micro electro-mechanical systems (MEMS) and complementary metal oxide semiconductor (CMOS) the characteristics of MEMS magnetometers, vertical Hall-effect devices, and bipolar magnetic transistors have been greatly improved [8,9,10,11,12]. Based on a heterojunction structure and a magnetic sensistive transistor with a long base region [13,14,15], a magnetically sensitive transistor with a nc-Si:H/c-Si heterojunction is presented in this paper, and the characteristics of magnetic sensitivity and temperature for the proposed transistor are also studied here.

## 2. Basic Structure and Operating Principle

### 2.1. Basic Structure

**Figure 1:**
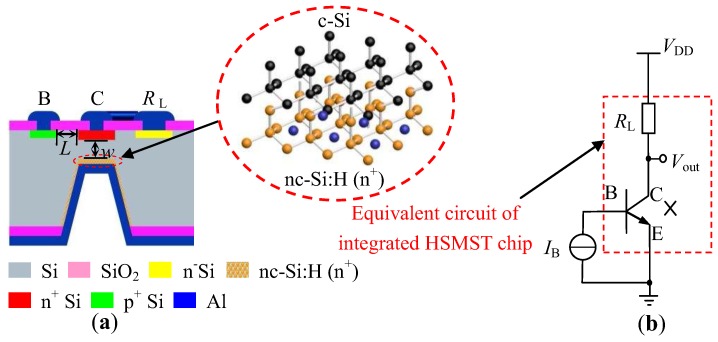
Basic structure and equivalent circuit of the heterojunction silicon magnetically sensitive transistor (HSMST): (**a**) Basic structure; (**b**) Equivalent circuit.

Figure 1a shows the basic structure model of an integrated heterojunction silicon magnetically sensitive transistor (HSMST) chip. The proposed integrated chip is constructed by a nc-Si:H/c-Si heterojunction magnetic sensitivity transistor and an integrated load resistor ($R_{L}$). The HSMST contains an emitter (E), a base (B), and a collector (C). *L* is the length of the base region for HSMST, and *w* is the width of the base region. The inset shows the atom distribution at the interface of nc-Si:H thin films and silicon substrate, where a heterojunction structure is formed. Figure 1b shows the equivalent circuit of the HSMST testing circuit, where $V_{\mathrm{DD}}$ is the supply voltage, $I_{B}$ is the base current, $R_{L}$ is the collector load resistor, and $V_{\mathrm{out}}$ is the output voltage of the chip. As shown in Figure 1b, the part in the dashed box is the equivalent circuit for integrated HSMST chips.

### 2.2. Operating Principle

#### 2.2.1. Heterojunction Transistor

**Figure 2:**
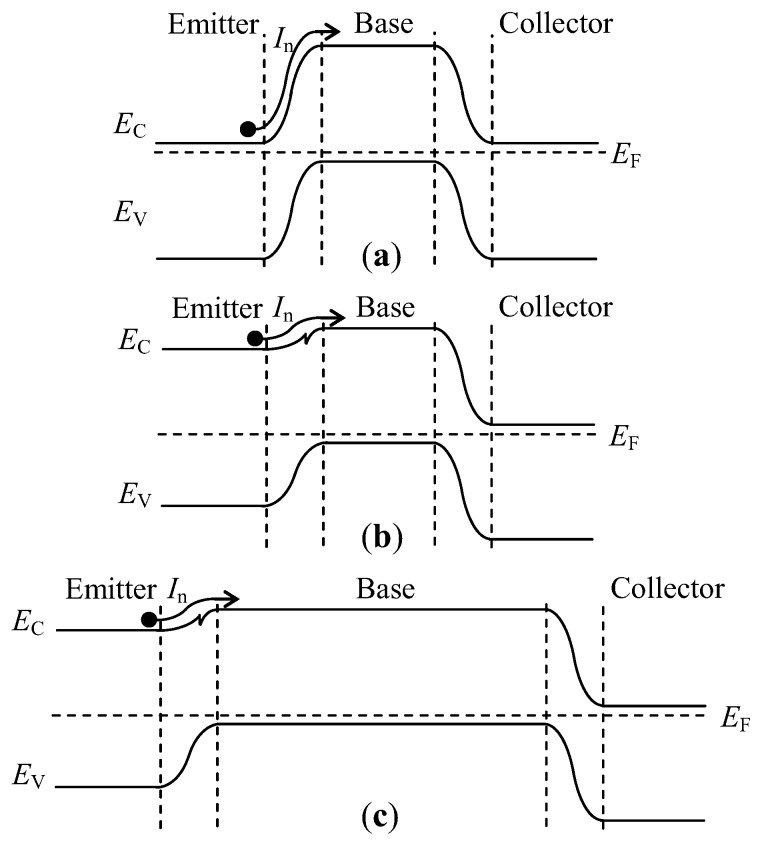
Transistor band diagrams: (**a**) Band diagram of the homojunction transistor; (**b**) Band diagram of the heterojunction transistor; (**c**) Band diagram of the HSMST with a long base region.

Figure 2a–c show the band diagrams of a homojunction transistor, a heterojunction transistor, and an HSMST, respectively. As shown in Figure 2b, the bandgap width for the heterojunction emitter junction is obviously improved compared with the band diagram of the homojunction transistor in Figure 2a. The carrier injection efficiency (*γ*) from the emitter junction is a significant figure of merit for a transistor, and it is defined as in Equation (1) [16].

(1) $$\gamma =\frac{I_{\mathrm{nE}}}{I_{\mathrm{nE}}+I_{\mathrm{pE}}}=\frac{1}{1+I_{\mathrm{pE}}/I_{\mathrm{nE}}}$$

where $I_{\mathrm{pE}}$ is the hole diffusion current to inject into the emitter from the base region, and $I_{\mathrm{nE}}$ is the electron diffusion current to inject into the base region from the emitter.

In the homojunction transistor, $I_{\mathrm{pE}}$ is much larger than the current lost by recombination in the base, mainly because of the bandgap shrinkage in the emitter, which causes the injection efficiency to be smaller. The heterojunction transistor creates a large barrier for the hole to inject into the emitter, utilizing the bandgap difference of the emitter and the base; therefore, this heterojunction structure will increase *γ* [16]. Differing from general heterojunction transistors, the proposed HSMST has a long base region, and the band diagram of the HSMST is shown in Figure 2c. The heterojunction structure produces a higher emitter injection efficiency, and the long base region generates magnetically sensitive characteristics, so the structures enhance the magnetic sensitivity of the HSMST.

#### 2.2.2. Magnetic Sensitivity

**Figure 3:**
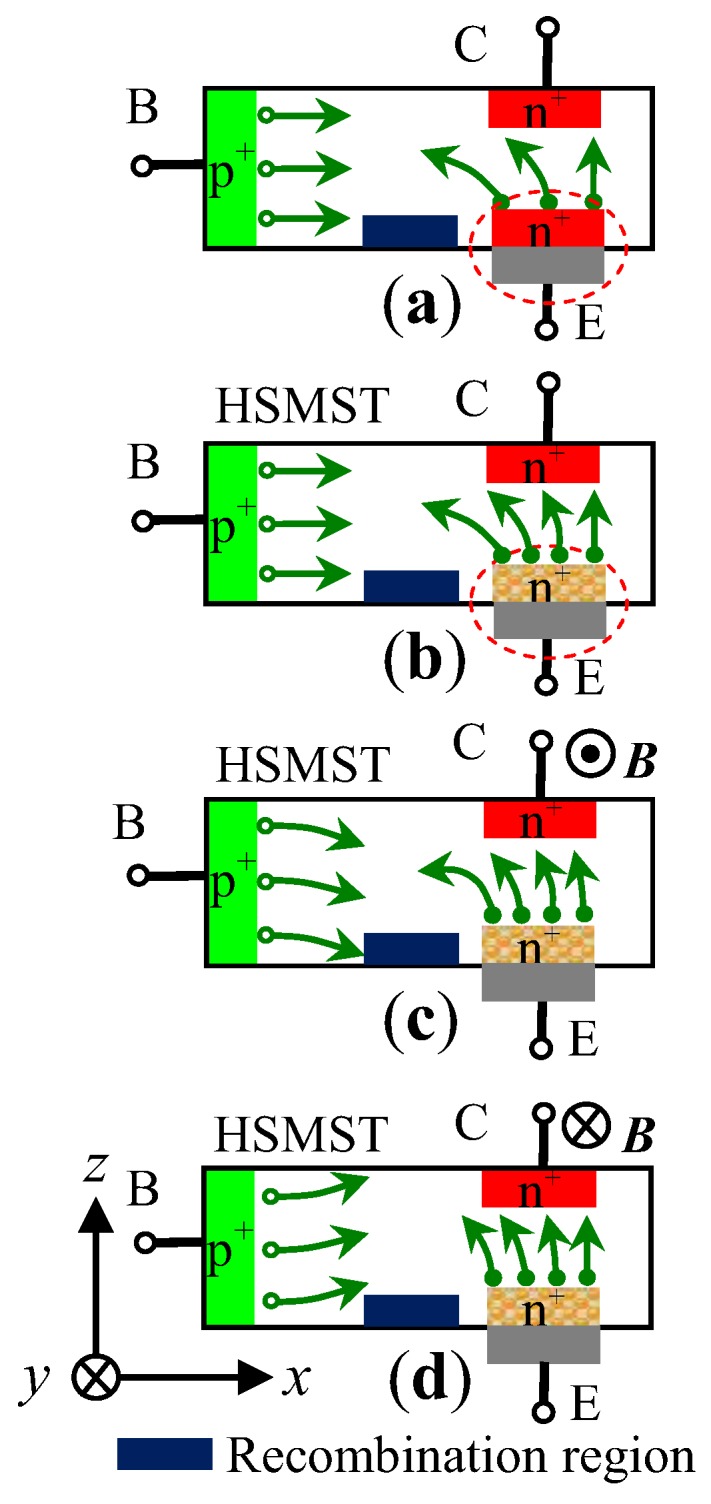
The working principle of the magnetically sensitive transistor (MST) under different *B*: (**a**) *B* = 0 T; (**b**) *B* = 0 T; (**c**) *B* > 0 T; (**d**) *B* < 0 T. B: base; C: collector; E: emitter.

Figure 3 illustrates the working principle of a magnetically sensitive transistor (MST) under different external magnetic fields (*B*) along the direction of the *y*-axis. The movement of carriers for the homojunction magnetically sensitive transistor without an external magnetic field is shown in Figure 3a. Figure 3b shows the activity for HSMST in the absence of an external magnetic field. As shown in Figure 3c, when an external magnetic field (*B* > 0 T) is applied along the direction of the negative *y*-axis, the collector current is decreased. As one can observe in Figure 3d, under the magnetic field (*B* < 0 T) along the opposite *y*-axis direction, the variation of the collector current is reversed with respect to Figure 3c.

When acted upon by an external magnetic field along the direction of the *y*-axis, the carriers injected into the base region from the emitter junction with nc-Si:H/c-Si heterojunction are deflected by the Lorentz force, so it is possible that the collector current ($I_{C}$) changes with *B*. The magnetic sensitivity ($S_{C}$) of the collector current can be expressed as [17]:

(2) $$S_{C\pm }=\left|\frac{I_{C\pm }-I_{C0}}{B}\right|,$$

where $I_{C+}$ is the collector current under *B* > 0 T, $I_{C-}$ is the collector current under *B* < 0 T, and $I_{C0}$ is the collector current at *B* = 0 T.

According to the equivalent circuit in Figure 1b, the collector current is transformed to a collector output voltage by an integrated load resistor, so collector output voltage ($V_{\mathrm{out}}$) changes with *B*. The magnetic sensitivity ($S_{V}$) of the collector output voltage can be expressed as [17]:

(3) $$S_{V\pm }=\left|\frac{V_{\mathrm{out}\pm }-V_{0}}{B}\right|=\left|\frac{\Delta V}{B}\right|,$$

where $V_{\mathrm{out}+}$ is the collector output voltage under *B* > 0 T, $V_{\mathrm{out}-}$ is the collector output voltage under *B* < 0 T, $V_{0}$ is the collector output voltage at *B* = 0 T, and ΔV is the difference between $V_{\mathrm{out}\pm }$ and $V_{0}$.

According to Equations (2) and (3), it can be derived that each of $I_{C}$ and $V_{\mathrm{out}}$ changes with *B*. As a result, the detection of the magnetic field could be realized by the HSMST.

## 3. Fabrication Technology

**Figure 4:**
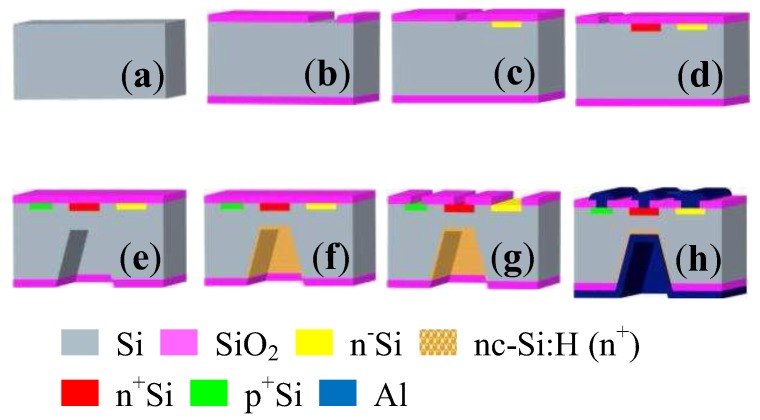
Main fabrication process of the integrated chip: (**a**) Cleaning wafer; (**b**) First photolithography; (**c**) Form the collector load resistor and second photolithography; (**d**) Making the collector and third photolithography; (**e**) Fabricating the base region and fourth photolithography; (**f**) Fabricating the emitter; (**g**) Fifth photolithography as a pin hole; (**h**) Sixth photolithography to form the electrodes.

Figure 4 shows the main processing steps of the integrated HSMST chips. (a) Cleaning a p-type <100> orientation silicon wafer with high resistivity; (b) growing a SiO_2_ layer with a thickness of 600 nm by thermal oxidation, and then first photolithography to etch a SiO_2_ layer as the window of the collector load resistor; (c) n^+^ type doping to form the collector load resistor and then depositing the SiO_2_ layer after clearing the wafer, a second photolithography to fabricate the window of collector; (d) n^+^ type heavily doping to make the collector and depositing the SiO_2_ layer after clearing the wafer, through a third photolithography to fabricate the window of the base region; (e) p^+^ type heavily doping to fabricate the base region, depositing the SiO_2_ layer after clearing the wafer, via a fourth photolithography to etch the lower surface as the window of the emitter and make a C shape silicon cup (C shape silicon cup is an etch pit) by ICP (inductively coupled plasma); (f) n^+^ type heavily doping to the lower surface of the C shape silicon cup to fabricate the emitter, and high-temperature annealing to wafer at 1000 °C for a half-hour; (g) the fifth photolithography to etch the upper surface as a pin hole; (h) metal Al made by vacuum evaporation and a sixth photolithography to form the electrodes, growing metal Al on the lower surface by vacuum evaporation, metallizing at 420 °C for twenty minutes to form an ohmic contact. The chips are fabricated on the p-type <100> double-sided polished silicon wafer with high resistivity by micro electro-mechanical systems (MEMS) technology and chemical vapor deposition (CVD) method.

**Figure 5:**
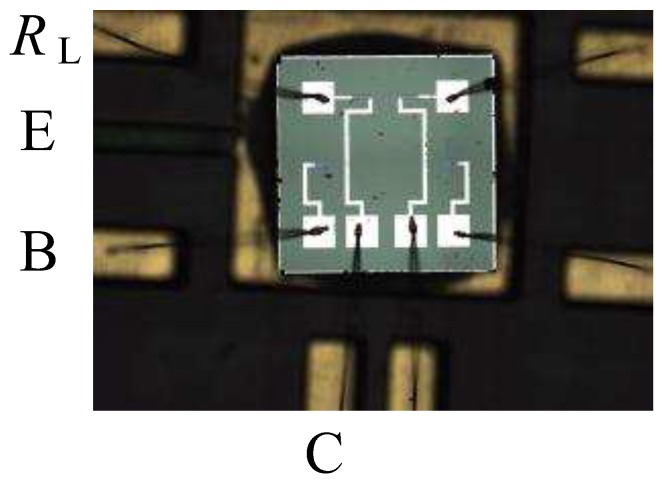
Packaging photograph of the integrated HSMST chip.

The chip is fixed on a printed circuit board (PCB) and then packaged by a bonder of integrated circuit inside wire. Figure 5 shows a photograph of the packaged integrated HSMST chip composed of a collector, a base, an integrated load resistor on the front-side, and an emitter on the back-side.

## 4. Results and Discussion

### 4.1. I–V Characteristics

**Figure 6:**
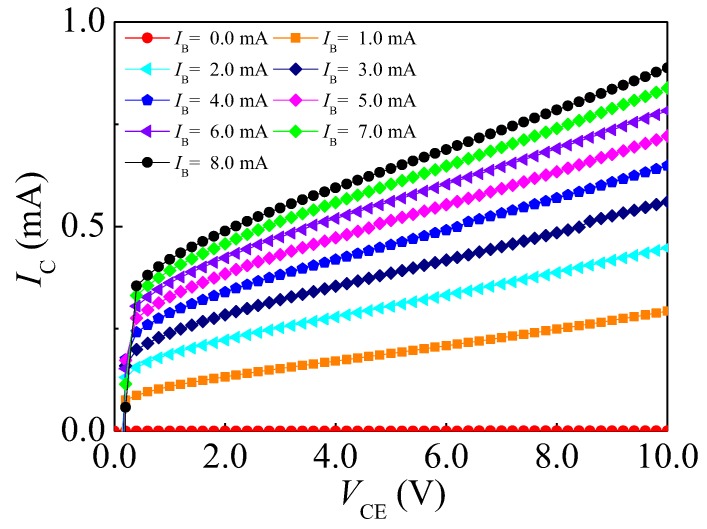
$I_{C}$–$V_{\mathrm{CE}}$ characteristic curves of the HSMST.

The current–voltage ($I_{C}$–$V_{\mathrm{CE}}$) characteristics of the proposed transistors were measured by a semiconductor characteristic test system (KEITHLEY 4200, Keithley, Cleveland, OH, USA). The $I_{C}$–$V_{\mathrm{CE}}$ characteristics of the HSMST are shown in Figure 6. When *B* = 0 T, the base current ($I_{B}$) changes from 0 to 8.0 mA with steps of 1.0 mA, and $V_{\mathrm{CE}}$ changes from 0 to 10.0 V with steps of 0.2 V. At a constant $I_{B}$, the $I_{C}$ increases with $V_{\mathrm{CE}}$. The $I_{C}$–$V_{\mathrm{CE}}$ curves become flat for the homojunction transistor, while $V_{\mathrm{CE}}$ reaches a certain value. In this paper, the $I_{C}$–$V_{\mathrm{CE}}$ curves do not become flat, the proposed transistor has unsaturation characteristics. When $V_{\mathrm{CE}}$ is fixed, the $I_{C}$ is less than the $I_{B}$, so the current amplification coefficient (*β*) of the HSMST is less than 1. In this instance, the *β* is unequal at different $I_{B}$.

### 4.2. Temperature Characteristics

**Figure 7:**
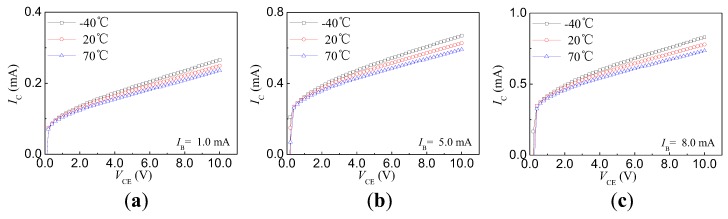
Temperature characteristics of the magnetic sensitivity transistor with nc-Si:H/c-Si heterojunction: (**a**) $I_{B}$ = 1.0 mA; (**b**) $I_{B}$ = 5.0 mA; (**c**) $I_{B}$ = 8.0 mA.

The $I_{C}$–$V_{\mathrm{CE}}$ characteristics at different temperatures were measured by a high- and low-temperature humid chamber (GDJS-100 LG-G, OBIS, Suzhou, China). Figure 7 indicates the $I_{C}$–$V_{\mathrm{CE}}$ characteristics of HSMST at different temperatures, including −40°C, 20°C, and 70°C. The temperature characteristics of HSMST when $I_{B}$ = 1.0 mA, 5.0 mA, and 8.0 mA are shown in Figure 7a–c, respectively. The experimental results show that $I_{C}$ has a negative temperature coefficient, as shown in Figure 7. The carriers injected from the emitter junction are divided into two cases. The carriers passing through base region are collected by the collector region, and the carriers are recombined in the base region. The number of carrier recombinations increases with temperature. Under constant $V_{\mathrm{CE}}$ and $I_{B}$, the $I_{C}$ decreases with the temperature.

Temperature coefficient ($\alpha_{C}$) of the collector current can be expressed as [17]:

(4) $$\alpha_{C}=\frac{I_{C}\left(T_{2}\right)-I_{C}\left(T_{1}\right)}{I_{C}\left(T_{0}\right)\left(T_{2}-T_{1}\right)}\times 100\%/{\;}^{\circ }C$$

where $I_{C}$($T_{2}$), $I_{C}$($T_{1}$), and $I_{C}$($T_{0}$) are the collector current at $T_{2}$, $T_{1}$, and room temperature, respectively.

**Figure 8:**
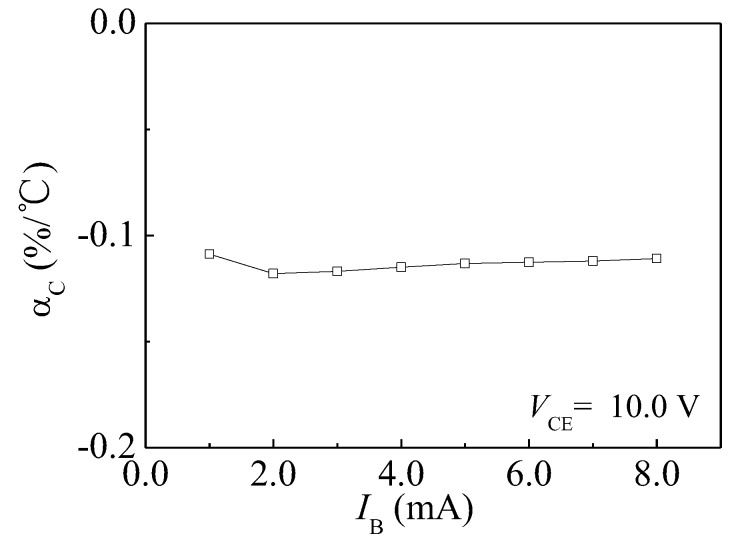
The relationship curve between $\alpha_{C}$ and $I_{B}$.

The $\alpha_{C}$ is calculated according to Equation (4). When $I_{B}$ = 8.0 mA and $V_{\mathrm{DD}}$ = 10.0 V, the $\alpha_{C}$ is −0.11%/°C. The calculated results demonstrate that the $\alpha_{C}$ of HSMST is a negative temperature coefficient. Figure 8 shows the relationship curve between $\alpha_{C}$ and $I_{B}$. When $V_{\mathrm{CE}}$ = 10.0 V, the $\alpha_{C}$ remains approximately constant. However, $I_{C}$ has a smaller temperature coefficient at $I_{B}$ = 2.0 mA. The temperature drift gradually increases with $I_{B}$ when $I_{B}$ < 2.0 mA.

### 4.3. Magnetic Sensitivity Characteristics

**Figure 9:**
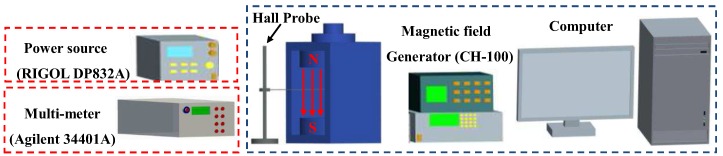
Testing system of the magnetic field sensor.

As shown in Figure 9, the testing system of the magnetic field sensor includes a magnetic field generator (CH-100, Beijing Cuihaijiacheng Magnetic Technology (Beijing, China), a multi-meter (Agilent 34401A, Agilent, Santa Clara, CA, USA), a power source (RIGOL DP832A, RIGOL, Beijng, China), and a computer.

When the sensor was acted upon by a constant external magnetic field in the range −0.6 T ≤ *B* ≤ 0.6 T controlled by a computer, the magnetic characteristics of HSMST at room temperature could be measured.

#### 4.3.1. Current Magnetic Sensitivity

**Figure 10:**
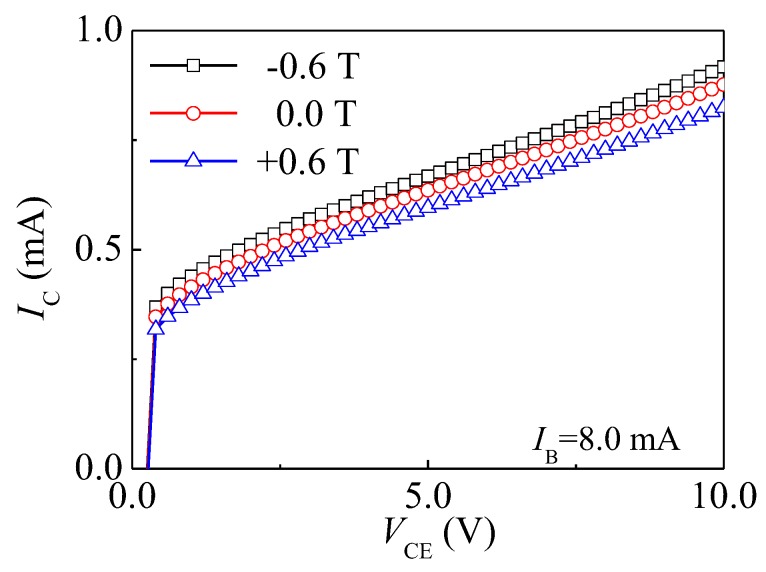
$I_{C}$–$V_{\mathrm{CE}}$ characteristics of the HSMST under different *B*.

**Figure 11:**
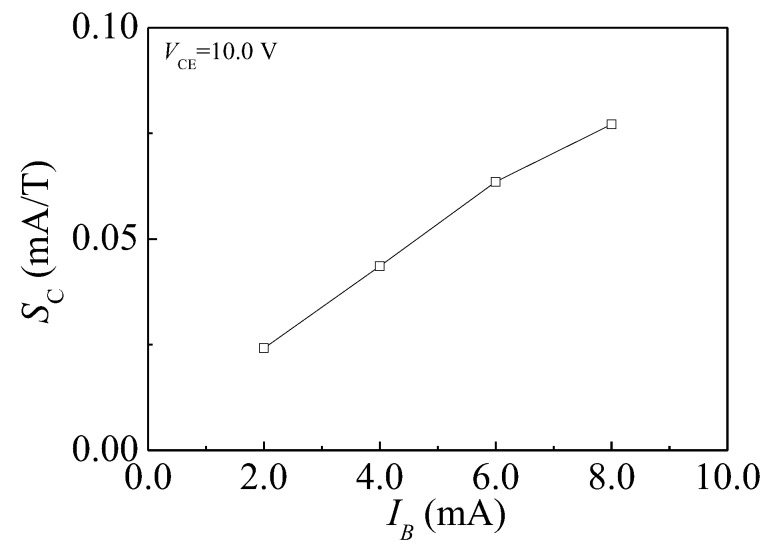
The relationship curve between $S_{C}$ and $I_{B}$.

Figure 10 shows $I_{C}$–$V_{\mathrm{CE}}$ characteristics of the HSMST with $I_{B}$ = 8.0 mA at different *B*. On the condition of constant $V_{\mathrm{CE}}$ and $I_{B}$, the $I_{C}$ has a minor value at *B* = +0.6 T and a major value at *B* = −0.6 T. Based on Equation (2), the relationship curve between $S_{C}$ and $I_{B}$ is plotted as shown in Figure 11 through numerical calculations, when $V_{\mathrm{CE}}$ = 10.0 V and the $I_{B}$ is from 2.0 to 8.0 mA with steps of 2.0 mA. When $I_{B}$ = 8.0 mA, the $S_{C}$ is 0.077 mA/T. On the condition of constant $V_{\mathrm{CE}}$, $I_{C}$ increases with $I_{B}$, and the $S_{C}$ is enhanced with $I_{C}$. At $V_{\mathrm{CE}}$ = 10.0 V, the $S_{C}$ increases with $I_{B}$.

#### 4.3.2. Voltage Magnetic Sensitivity

**Figure 12:**
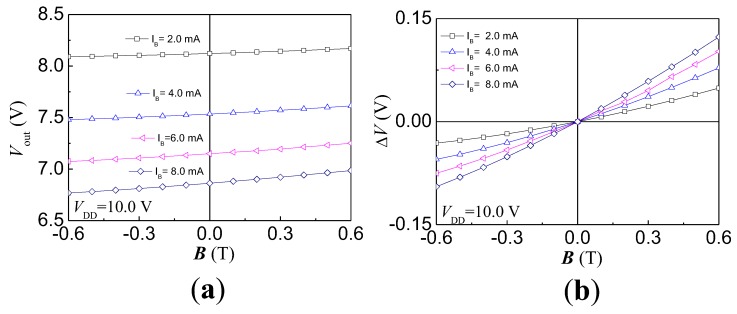
The magnetic characteristic curves of the HSMST chip: (**a**) Between $V_{\mathrm{out}}$ and *B*; (**b**) Between ΔV and *B*.

**Figure 13:**
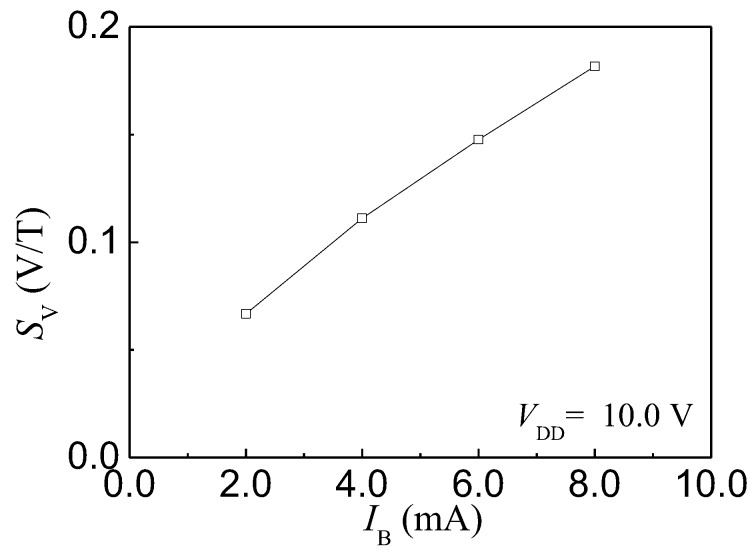
The relationship curve between $S_{V}$ and $I_{B}$.

The HSMST chip integrated a collector load resistor ($R_{L}$) of 4.1 kΩ. As shown in Figure 1b, the $I_{C}$ is converted to $V_{\mathrm{out}}$ by $R_{L}$. Figure 12a shows the relationship curves between $V_{\mathrm{out}}$ and *B* with $V_{\mathrm{DD}}$ = 10.0 V at different $I_{B}$. When both $V_{\mathrm{DD}}$ and $I_{B}$ are constant, $V_{\mathrm{out}}$ increases with *B*. The $V_{\mathrm{out}}$ decreases with $I_{B}$ under constant $V_{\mathrm{DD}}$ and *B*. According to the relationship of ΔV, $V_{\mathrm{out}\pm }$ and $V_{0}$, the relationship curves between ΔV and *B* are plotted as shown in Figure 12b by numerical calculations when $V_{\mathrm{DD}}$ is 10.0 V and $I_{B}$ is from 2.0 to 8.0 mA, with steps of 2.0 mA. The ΔV increases with *B* at constant $V_{\mathrm{DD}}$ and $I_{B}$. Under constant $V_{\mathrm{DD}}$ and *B*, ΔV increases with $I_{B}$. $S_{V}$ is calculated based on Equation (3), and the relationship curve between $S_{V}$ and $I_{B}$ is shown in Figure 13. At room temperature, $I_{B}$ = 8.0 mA and $V_{\mathrm{DD}}$ = 10.0 V, the $S_{V}$ is 181 mV/T. Under constant $V_{\mathrm{DD}}$ and *B*, ΔV increases with $I_{B}$, and the $S_{V}$ increases with ΔV. When $V_{\mathrm{DD}}$ = 10.0 V, the $S_{V}$ increases with the $I_{B}$.

## 5. Conclusions

In summary, a magnetically-sensitive transistor with a nc-Si:H/c-Si heterojunction is presented, where the integrated HSMST chips were designed and fabricated by adopting MEMS technology and CVD method. The magnetic and temperature characteristics of the HSMST are studied in this paper. The experimental results show that the HSMST has unsaturation characteristics, and the current amplification coefficient (*β*) is less than 1. When $I_{B}$ = 8.0 mA and $V_{\mathrm{DD}}$ = 10.0 V, the magnetic sensitivity and the temperature coefficient of HSMST are 181 mV/T and −0.11%/°C, respectively, which indicates the HSMST not only has superior magnetic sensitivity of positive and negative direction, but also good temperature characteristics. It is very important to improve the properties of magnetic sensors.
